# Phymosis with Impermiable Meatus Urinarius, Operation

**Published:** 1868-08

**Authors:** C. C. F. Gay

**Affiliations:** Buffalo, N. Y.


ART. III—*Phymosis with Impermiable Meatus Urinarius, operation. Intestinal Invagination, Strangulation, Sloughing and Discharge per rectum of fourteen inches of intestine, with fatal hemorrhage.* BY C. C. F. GAY, M. D., *Buffalo, N. Y.*

The first patient was a lad 14 years of age. How long the phymosis and impermiable urethra had existed could not be ascertained. It had been known for a long time by the mother that much difficulty in micturating existed. The patient had anasarca. Operation: Slit up the prepuce partially and found the prepuce adherent firmly to the gland in its entire circumference. The patient was now anæsthetized and the prepuce separated from the gland by tearing with the finger-nail and by frequent use of the knife; the operation consisted more of dissection than of tearing with the nail, and the cutting was found necessary over the entire surface of the glands penis, and was attended by considerable hemorrhage. Search was now made for the meatus, but in vain; made an incision at the point where we thought the meatus ought to be, and was enabled to introduce a small silver probe into the urethra; the urethra was afterwards dilated by the bougie. Small pleggets of lint, wetted with glycerine, were packed firmly between the prepuce and gland, one layer upon another, for the purpose of preventing adhesion, and were removed in two days, and the same tamponing process repeated for a fortnight; at the expiration of three weeks the boy had recovered with relief of the phymosis and being able to pass a full stream of water.

There are three points of interest in this case; first, the patient was only able to pass a stream of water not much larger than that of a cambric-needle, and but little at a time with frequent desire. Anasarca had been observed for a long time, and must have been caused by the boy's inability to properly and fully evacuate the contents of the bladder; secondly, was this abnormal condition of the penis congenital? We are not able to answer this question satisfactorily. From the lad's previous history we were unable to learn either from the father or mother, or the boy himself, that there had ever existed inflammation of the organ to cause adhesion of the prepuce to the gland or to occlude the meatus; if there ever had been, the inflammation must have had an existence a long time prior to the operation, because of the firm adhesions, which could only be torn at points, so firm were they, and requiring the knife to separate them. A third point of interest connected with this case is the fact of the co-existence of the impermiable meatus and phymosis. This may be common and seem no novelty to other observers; possibly one of these conditions may predispose the patient to the other condition, and the one abnormity may have been depended upon the other.

The boy was operated upon April 20th, 1868, with relief of the phymosis and impermiable meatus, and with this relief his anasarca has disappeared.

Mr. S., aged 49 years, previous good health, suddenly fell ill, with pain in the right hypogastrium and rigors, which were attributed to a cold. After lying in bed for a day or two, got up in the night at the call of the alarm fire-bell and stood out upon the side-walk, barefooted, for some time, and became much chilled; returning to his room and getting warm, the abdominal pain became intense, and never quite left him during an illness of three weeks. His symptoms were unusual, but peculiar to the disease under which he was suffering, and so prominent and well marked as to enable one to correctly diagnose intestinal invagination. It is, therefore, on account of their diagnostic value that the symptoms are detailed and the case reported.

The most prominent symptoms, aside from those which usually accompany enteritis were the following: complete retention of urine, constipation of bowels at first, afterwards diarrhœa, frequent syncope, inability to maintain the recumbent posture, the semi-recumbent posture insisted upon by the patient during the entire illness, profuse diaphorisis and continuous rigors, pulse never reaching beyond 100 per minute until the last day of illness, pain and tenderness so great that anodynes used hypodermically, and by the stomach and rectum, could but imperceptibly abate; two grains of morphine used sub-dermily in the course of an hour, with one grain given by the stomach and two thrown into the rectum, would scarcely abate the intensity of the suffering.

On the eighteenth day, on examining the rectum, there was seen protruding about one inch of what looked to be a thick fold of mucous membrane discolored. Two days thereafter, Dr. Storck, with whom I was associated in the treatment of the case, finding this foreign body protruding considerably further through the anus, seized hold of it, and with the slightest traction it was made to pass away, but the doctor remarked that before it all came away it seemed to him that the entire length of the intestines were passing from the patient—fourteen inches of intestine thus passed away, and was followed by fatal hemorrhage, the patient surviving the loss of this amount of intestine twenty-four hours. I made trial to preserve the morbid specimen in order to present it to the Association, but was unable to preserve it in alcohol. At two points it maintained its cylindrical shape, but the greater portion of it was broken down and preserved only a shapeless, gangrenous mass.

The following query is suggested by this case, viz: Could it be possible for a patient to recover after fourteen inches of his intestine had sloughed away? This question has been satisfactorily answered by pathologists in the affirmative. Cases are on record and well authenticated, wherein a greater extent of intestine than this reported has passed per rectum, the patient making a good recovery. Union between the two extremities of intestine having taken place the continuity of the alimentary canal has been re-established and the patient's life thus preserved.

A report was made in July, 1855, upon this very subject before the Academy of Medicine of Paris, and after full discussion the report was adopted by the Academy. The cases reported and the discussion which followed may be found published in the *Bulletin de L'Académie Impériale de Medicine* for 1855, from which I take the liberty of quoting a single paragraph:

"La science possède de nombreux examples de faits analogues à celui que nous présentons en ce moment à l'académie. Il serait trop long d'en faire ici l'inumération complete. Qu'il nous suffice d'en citer un certain nombre dont le caractére anatomique a été reconnee de la maniére la plus incontestable par des anatomistes et des chirugiens dont les connaissances ne sauraient étre un moment révoquées en donte."

